# Status of the Colored Brother in Relation to the A. M. A.

**Published:** 1906-04

**Authors:** 


### STATUS OF THE COLORED BROTHER IN RELATION TO THE A. M. A.

*"Yes, colored physicians are eligible to membership in the American Medical Association, and there are colored physicians at present members of the American Medical Association."—Dr. Geo. H. Simmons, Secretary, and Editor Journal A. M. A.*

Worse and worse! We have practically agreed to swallow every other kind of doctor, and if we do not gag at osteos, homoes, physio-meds, and faith healers, the next figure on the program of the great Medical Union and Medical Journal Trust is to force the negro down our throats. Fancy sitting by Dr. Sam at an A. M. A. banquet!

The following correspondence will be read with deep interest by all Southern physicians, at least:

AUSTIN, TEXAS, March 8, 1906.

*Dr. Geo. H. Simmons, Secretary A. M. A., Chicago.*

DEAR DOCTOR: Having been interrogated by some of my colleagues of the Texas State Medical Association as to the status of colored physicians in relation to the American Medical Association, I beg to ask of you information on the subject for publication. Please see page 333 of the TEXAS MEDICAL JOURNAL for March. I there quote the *Medical Sentinel* to the following effect: "The American Medical Association declares that any practitioner of medicine who is a gentleman, regardless of school of practice, may become a member of that body." * * * "The American Medical Association has delegated to that body (meaning the county medical societies) the privilege of naming those who may or may not become members of the parent organization, stipulating only that no discrimination shall be exercised against candidates merely because schools of practice differ."

That is to say, if I understand it, any physician of any school, "Regular," Homeopath, Eclectic, Osteopath, Physio-Medical, or what not, *black* or white, may become a member of the National Association, provided he is "a gentleman." Now, does the American Medical Association say that a colored physician is not a gentleman, and discriminate against him on account of race color or previous condition of servitude? If so, I am answered, and no negro need apply. If otherwise, the negro of any school it would seem is eligible; if not, why not? Please answer. In Texas, he is not admitted to state or county society. In other States doubtless he is, and my specific inquiry is: Are colored physicians eligible to membership in the American Medical Association, and are there any colored physicians at present members of the American Medical Association?

The *Medical Sentinel* further says: "The National body officially represents the entire profession." Are the colored brethren a part of the profession, and are they represented?

Awaiting your official answer, I am, sir,

Respectfully yours,
F. E. DANIEL, M. D.,
Editor TEXAS MEDICAL JOURNAL.

AMERICAN MEDICAL ASSOCIATION.
OFFICE OF GENERAL SECRETARY.
103 DEARBORN AVE.

CHICAGO, March 13, 1906.

*Dr. F. E. Daniel, Austin, Texas.*

DEAR DOCTOR: Your letter of the 8th inst. has been received, and I am very glad to give you the information asked for. Your letter, analyzed, asks two questions: First, whether the American Medical Association admits Homeopaths, Eclectics, Physio-Medicals, Osteopaths, etc., and, second, whether it admits negroes.

First, in regard to the admission of sectarians, I enclose a copy of the Constitution and By-Laws, and quote from the section governing membership: "A member in good standing of the constituent Association of the State in which he resides, or a medical officer of the U. S. army, of the U. S. navy, or of the U. S. Public Health and Marine Hospital Service, may become a member of the American Medical Association," etc. The second and third sections provide that when a member of the A. M. A. loses his membership in the lower society, or in the government service, he thereby loses his right to membership in the National Association. When he is restored to membership in his local society, he can again become a member of the A. M. A. In other words, membership in the A. M. A. depends on membership in the State Association, or one of its component branches.

The American Medical Association does not indicate to the State Association whom it may or may not admit. The State Association usually allows the component county society to say who shall be admitted to membership, subject, however, to certain limitations. For instance, the Constitution and By-Laws of the State Medical Association says:

> "Each county society shall judge of the qualification of its own members, but, as such societies are the only portals to this Association and to the American Medical Association, every reputable white and legally registered physician who is practicing, or will agree to practice, non-sectarian medicine, shall be entitled to membership."

This section appears in the By-Laws of most of the State Associations, modified in some instances by the omission of the word "white."

A reference to the By-Laws of the county societies will show that the same provision occurs there, showing that sectarians can not be admitted unless they give up their sectarian claims. For instance, Article III of the standard Constitution for county societies says:

> "Every legally registered physician residing and practicing in ..........county, who is of good moral and professional standing, and who does not support or practice, or claim to practice, any exclusive system of medicine, shall be eligible to membership."

Replying to your second question: "Are colored physicians eligible to membership in the American Medical Association, and are there any colored physicians at present members of the American Medical Association?" To both of these questions the answer is "yes." But you are bringing up a question that was settled many, many years ago. Let me quote from the address of Dr. J. Marion Sims, one of the greatest surgeons that this or any other country ever produced, which he delivered as president of the American Medical Association in 1876:

> "Who can forget the last meeting held in this city, Philadelphia, in 1872, when so much time was wasted in discussing the woman question and the negro question, incidentally arising out of the question of representation? Fortunately for us these vexed questions are now all settled quietly and in the most natural way. Formerly we admitted delegates from small corporations and local societies that were sometimes temporarily hatched up for the purpose of forcing objectionable persons upon us. Now we are a truly representative body, made up of delegates from State and county medical societies; and we must, of necessity, receive such delegates as any of these societies, properly organized, may see fit to send us."

The general principles as stated by Dr. Sims in 1876 apply today.

If the above does not cover the points you have in mind, let me hear from you again.

Respectfully yours,

GEORGE H. SIMMONS.

RAISE THE LONE STAR FLAG ONCE MORE.—This decision should cause every Southern physician to withdraw from this olla-padrida, this pot-pouri, where all is fish that come to the Octopus' net. The lion and the black lamb will never lie down together. Let the Texas State Medical Association *secede from the medical union,* and once more raise the *Lone Star Flag of Texas.*

---

HOMOEOPATHIC LEAVENING OF THE A. M. A. LOAF: *"Beware of the leaven of the Pharisees, which is hypocricy."*—(Luke xii, 1.) THE A. M. A. AS A LEVELER OF DISTINCTIONS: The *homoeopathic leaven is at work,* and medicine must be leveled to the plane of the pathies and the darkies. That is the "toot," as I understand it, that was sounded by Gabriel McCormack in the blast he gave the Oregon fellows. Indeed, when McCormack was here, in his lecture he advised us to blend with the homos, and notwithstanding the Constitution of the county medical societies distinctly says that professional recognition shall not be extended to those who practice upon an exclusive dogma, the councilman of the district and the Texas Tentacle of the Octopus at Fort Worth, are advocating it. It is time for self-respecting physicians to cut loose from the American Medical Association and refuse to be thus herded with the ring-streaked and striped cattle at the behest of the great reformed homo who now runs that machine. Miscegenation will be the next move.

---

THE STATE ASSOCIATION'S BILL for a mixed Board of Medical Examiners will be found in this issue. Of all the absurdities I ever saw, this is the greatest. It is a blunder. It is the logical outcome of that which has gone before (mentioned above), the leveling of all distinctions between regular medicine and the numerous "pathies" and the colored contingent. Possibly the scheme originated in Chicago. Possibly it was ordered. The desire to stand in with the Octopus crowd—to harmonize, to "unite the profession," has caused our able and distinguished Committee on Medical Legislation to take a step which, it seems to me, their better judgment and their pride of character as physicians would condemn. The Secretary of the Association, Dr. Chase, is ex-officio, a member of that committee. He informed me that he and ex-President Wilson—two of the five members—are opposed to it. The bill was submitted to every county society for action at the March meeting. If approved by a majority of county societies, it is to go before the House of Delegates at the April meeting of the State Association at Fort Worth, and if approved there, the committee will ask the Thirtieth Legislature to pass it and repeal the existing practice act. I have no means of knowing what action was had upon it at any county society meeting except our own. Here it was most emphatically condemned and our delegate instructed to oppose it.

IF THE LEGISLATURE RECOGNIZES all the pathies, as it has already done with regard to homos and eclectics, and as it surely will do next session by creating at least two more separate boards (osteos and physio-meds) that carries with it no obligation upon the medical profession to recognize them; and for the organized profession of Texas to voluntarily extend to them an invitation to sit on a medical board with its own members, is a stultification, a coming down, a lowering of the standard of medicine, a surrender of every principle of medical ethics, and a going back on the best and noblest traditions of the profession. Besides this, it is inexpedient. It will not stand a ghost of a chance to pass. The drugless element who are now, under the existing law, exempt from all restrictions, and may practice medicine, surgery and obstetrics,—provided they give no drugs or medicines,—are to be restricted from practicing surgery and obstetrics; and all who have been in practice since January, 1905, are to register, pay a fee, and receive a curtailed license. Does any one suppose that they will submit to it? They will fight it with more vigor than they did the amendment proposed last session, and will surely defeat it. The sentiment of the last Legislature was *pronounced* in favor of separate boards for all these fellows. Is it reasonable to suppose that the osteopaths, for instance, will consent to an arrangement that will give them one member on a board of ten? The bill makes no exception for the older physicians who qualified under old laws and have practiced many years, but requires them to show again their authority to practice, register *again,* and pay a fee. What are you old fellows going to do about it?

---

IT MAY BE THAT THE IDEALS of the old school of medicine, instilled into the writer who, like Gamaliel at the feet of Paul, sat under the teachings, of the great Fenner and the great Stone and the great Austin Flint, are too high for this commercial age, when everybody and everything called "doctor" can join the great national Association by paying $5. It may be so. But I will never lower the standard raised by the "Red Back" nearly a quarter-century ago and to which standard a strict conformity by the profession of Texas has ever been urged. It is a hard matter to teach old dogs new tricks; I can't quite come down to the level of pathies and negroes as brother physicians, and I have the courage, or shall I say indiscretion, to say so? And in saying so, I wish my readers to clearly understand that at a sacrifice of my interests, perhaps, I am urging resistance to the degradation of boss rule solely because I love the medical profession and have no higher ambition than to see it kept pure and uncontaminated and true to its traditions as exemplified in the lives of our truly great, the highest type of the American physician.

---

AN EXPERIENCED PRESCRIPTION PHARMACIST and hospital steward, licensed and registered, desires a position. Married, steady, capable and industrious. Best of references. Address, R. P. Daniel, 521 N. Frio St., San Antonio, Texas.

